# Estrogen-Mediated Neural Mechanisms of Sex Differences in Burning Mouth Syndrome

**DOI:** 10.3390/neurolint17040061

**Published:** 2025-04-20

**Authors:** Takahiko Nagamine

**Affiliations:** 1 Department of Psychiatric Internal Medicine, Sunlight Brain Research Center, Yamaguchi 747-0066, Japan; tnagamine@outlook.com; Tel.: +81-3-3726-1111; 2 Graduate School of Medical and Dental Sciences, Institute of Science Tokyo, Tokyo 113-8510, Japan

**Keywords:** burning mouth syndrome, default mode network, descending pain inhibitory pathway, estrogen, salience network, sex difference

## Abstract

Background/Objectives: Burning mouth syndrome (BMS) is a chronic pain disorder of the oral cavity in the absence of organic disease and is prevalent among menopausal women. Estrogen may be involved in the formation of nerves involved in pain. Methods: This paper presents an inferred mechanism for the relationship between estrogen and BMS based on a synthesis and interpretation of findings from a selection of published studies. Results: Estrogen influences the formation of neural circuits in BMS by dividing the complex pain circuit into the following three components: the peripheral pain circuit, brain network pain circuit, and memorized pain circuit. Conclusions: The development of BMS may be influenced by the formation of neural circuits by sex hormones.

## 1. Introduction

Burning mouth syndrome (BMS) is characterized by chronic, intractable, orofacial pain, defined as a burning or abnormal sensation in the mouth that occurs daily and recurs, lasts more than 2 h per day, lasts for more than 3 months, and has no clinically evident causative lesion on examination or investigation [1]. The prevalence of BMS has been observed to be higher in women compared to men, particularly in peri- and menopausal women, as evidenced by population-based studies [2], systematic reviews [3], and meta-analyses [4]. Estrogen levels fluctuate throughout a woman’s life, and changes can lead to various symptoms and conditions. In this paper, we examine the mechanism by which estrogen contributes to the formation of pain neural circuits in the development of BMS.

## 2. Methods

This paper presents an inferred mechanism for the relationship between estrogen and BMS based on a synthesis and interpretation of findings from a selection of published studies. Unlike a systematic review, this work does not aim for exhaustive coverage of all literature on the topic. Instead, it strategically draws upon key studies that provide relevant insights into the underlying processes. Studies were included if they directly investigated or provided substantial evidence related to effects of estrogen on the formation of pain neural circuits. We considered studies employing a range of methodologies, including in vitro experiments, in vivo models, and observational studies, to capture different perspectives on the phenomenon. Initial literature searches were conducted using relevant keywords in databases such as PubMed and Web of Science. The search strategy employed Boolean operators to combine the following keywords: ‘burning mouth syndrome’ AND ‘neural circuits’ AND ‘estrogen’ AND ‘mechanism’. We also followed citations from key articles to identify additional relevant studies. Selected papers were carefully read to identify key findings, experimental setups, and reported limitations. We focused on extracting information pertinent to the potential interactions and causal relationships involved in the effects of estrogen on the formation of the following three pain circuits.

## 3. Three Pain Neural Circuits of BMS

**Figure 1:**
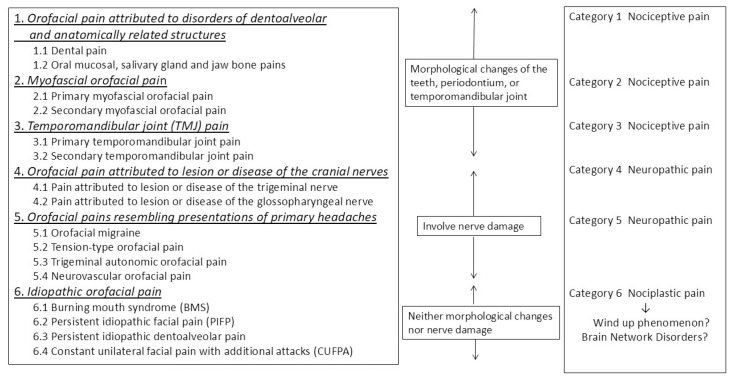
The position of burning mouth syndrome in the International Classification of Orofacial Pain (ICOP) classification (Quoted from reference [7] with some modifications).

The International Classification of Orofacial Pain (ICOP) system is a taxonomy that categorizes orofacial pain into six distinct categories, with category 6 corresponding to chronic orofacial pain, including BMS [1]. In contrast, the pathological classification of pain has recently been divided into three distinct categories: nociceptive pain, neuropathic pain, and nociplastic pain [5,6]. ICOP category 6, which encompasses BMS, is classified as nociplastic pain due to the absence of morphological changes, nociceptor stimulation, or neuropathy (Figure 1) [7]. Nociplastic pain is defined as pain that arises from altered nociception despite the absence of clear evidence of actual or threatened tissue damage causing the activation of peripheral nociceptors or evidence of disease or lesions in the somatosensory system causing the pain [8]. While the precise mechanisms underlying nociplastic pain remain to be fully elucidated, enhanced pain and sensory processing, along with altered pain modulation in the brain, are considered to be involved, including a wind-up phenomenon at the level of pain transmission in the spinal cord or altered processing of pain [9].

To understand nociplastic pain, it is useful to first define the components of the pain neural circuitry based on the definition of pain. According to the International Association for the Study of Pain (IASP), pain is defined as follows: “An unpleasant sensory and emotional experience associated with, or resembling that associated with, actual or potential tissue damage” [10]. This definition underscores the subjective nature of pain, emphasizing that it does not merely signify a physiological response to tissue damage. According to the aforementioned definition, the experience of pain comprises three distinct components: (1) sensation (nociception), (2) perception (interpretation), and (3) experience (memory). Each of these components is associated with its own brain circuit. The sensory component is the ascending pain transmission pathway that originates at peripheral pain receptors, traverses the dorsal horn of the spinal cord, the trigeminal nucleus, and the thalamus, and terminates in the primary somatosensory cortex of the brain. Here, it is regulated by the descending pain inhibitory pathway, which inhibits the transmission of pain stimuli to the spinal cord. These pathways that modulate and transmit pain to the cerebral cortex are called peripheral pain circuits [11]. Although the spinal cord is part of the central nervous system, it can be conceptualized as a highly complex transitional region; therefore, for practical purposes in this paper, we have conceptualized the subspinal regions as “peripheral” in terms of the transmission of pain stimuli. The perception element facilitates the transmission of signals from the primary somatosensory cortex to higher association cortices (e.g., the temporal lobe, occipital lobe, parietal lobe, etc.). The formation of emotions, meanings, and autonomic reflexes in response to pain occurs through several neural areas, which can be referred to as brain network pain circuits [11]. The experience component involves the evocation of information about pain or inflammation stored in the brain, regardless of the presence or absence of novel noxious stimuli, and is referred to as memorized pain circuits [11]. Modulation of neurotransmission in any of the neural circuits results in nociplastic pain. Nociplastic pain is known to involve all neural circuits from nociceptors to pain processing. Considering the mechanisms by which women are at risk in each pain circuit—the peripheral pain circuit responsible for pain perception, the brain network pain circuit responsible for pain recognition, and the memorized pain circuit that reproduces past pain memories even in the absence of painful stimuli—can help understand the sex differences in the pathogenesis of BMS.

## 4. Sex Differences in the Peripheral Pain Circuit

### 4.1. Nociceptors Expression

Research findings have indicated the presence of sex differences in the expression of pain receptors and the descending pain inhibitory pathway, which plays a crucial role in the suppression of pain. While sex hormones have been identified as contributing factors to the development of certain sex differences, it is a misconception to assert that estrogen exerts the most significant influence on these differences. Estrogen displays a dual effect on the nervous system, both inducing and alleviating pain, thereby regulating the sensation of pain. It is noteworthy that BMS also manifests during menopause, a period characterized by a decline in estrogen levels, rather than an increase. The involvement of estrogen is not linear but rather occurs in two stages: when estrogen levels increase, pain sensitivity increases, and when estrogen levels decrease, pain occurs [12].

The mechanism by which estrogens exert their effect on the promoter of the transient receptor potential cation channel subfamily vanilloid member 1 (TRPV1) receptor gene has been demonstrated to occur through both genomic and non-genomic pathways. This results in the transcription of the TRPV1 gene and an increase in TRPV1 production [13]. In addition to estrogens, prolactin has been demonstrated to increase TRPV1 receptor expression in dorsal root ganglion in female mice, a phenomenon that is not observed in male mice [14]. These findings suggest a potential link between heightened pain sensitivity during periods of elevated female hormone levels and the onset of BMS. As a primary hormonal influence, increased estrogen during puberty has been shown to elevate TRPV1 expression, rendering susceptible individuals more susceptible to pain and exacerbating pain responses to dental treatment. Indeed, patients with BMS may experience migraine headaches associated with TRPV1 and neuroinflammation prior to the manifestation of BMS [15].

**Figure 2:**
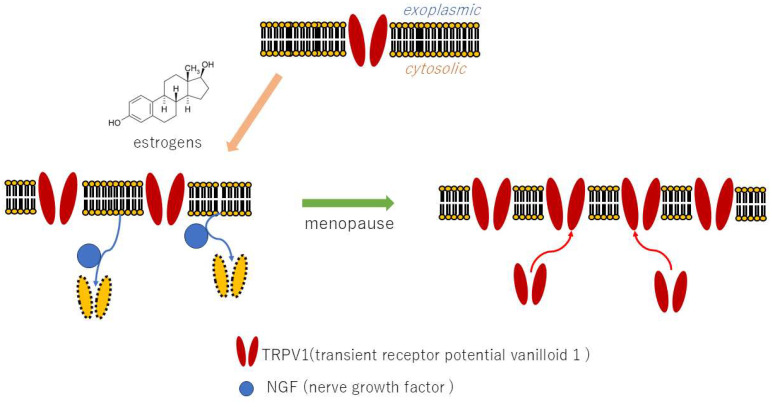
Effects of estrogen on TRPV1 gene expression and NGF.

In contrast, estrogens have been demonstrated to mitigate pain by modulating the expression of nerve growth factor (NGF). NGF facilitates the migration of TRPV1 to the cell surface membrane, thereby inducing a painful response. During menopause, a decline in estrogen levels occurs, resulting in the appearance of TRPV1 on the cell surface and contributing to the development of BMS [16]. A secondary impact of estrogen decline during menopause involves an increase in NGF, leading to an escalation in TRPV1 expression at the cell surface, an escalation in pain sensitivity, and the onset of neuroinflammation (Figure 2). This sequence of events contributes to the development of BMS in susceptible individuals. Indeed, studies have documented an augmentation in TRPV1 expression within the oral mucosa of BMS patients [17].

### 4.2. Wind-Up Phenomenon

**Figure 3:**
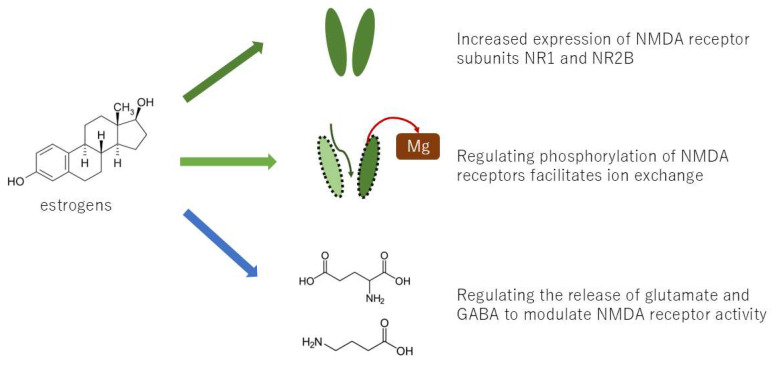
Several mechanisms by which estrogen causes the wind-up phenomenon.

The wind-up phenomenon in pain transmission at the spinal cord level is one of the mechanisms of nociplastic pain. The wind-up phenomenon is predominantly facilitated by NMDA (N-methyl-D-aspartate) receptors. Repeated stimulation of C-fibers has been demonstrated to result in increased glutamate release, which, in turn, activates NMDA receptors on spinal cord neurons. Research has demonstrated that estrogen can modulate the function and expression of NMDA receptors. Specifically, estrogens have been observed to potentiate glutamate responses, primarily through the enhancement of NMDA receptor activity, and also by affecting GABA-ergic mechanisms and altering brain morphology by increasing dendritic spine density [18]. Furthermore, estrogen has been demonstrated to enhance the processing of visceral nociception within the spinal cord by increasing NMDA receptor NR1 subunit expression and site-specific receptor phosphorylation on the NR1 subunit, thereby augmenting NMDA receptor activity [19]. Consequently, the likelihood of wind-up phenomenon occurrence is higher in women compared to men (Figure 3). Conversely, estrogen-induced analgesia has been documented, and it has been proposed that estrogen analgesia is peripherally mediated through non-genomic signaling mechanisms, with the decline in estrogen levels at menopause enhancing peripheral pain transmission [20]. The role of estrogens in nociception is organ-specific, exhibiting either pro- or anti-nociceptive properties depending on the specific organ involved.

### 4.3. Sex Differences in the Descending Pain Inhibitory Pathway

**Figure 4:**
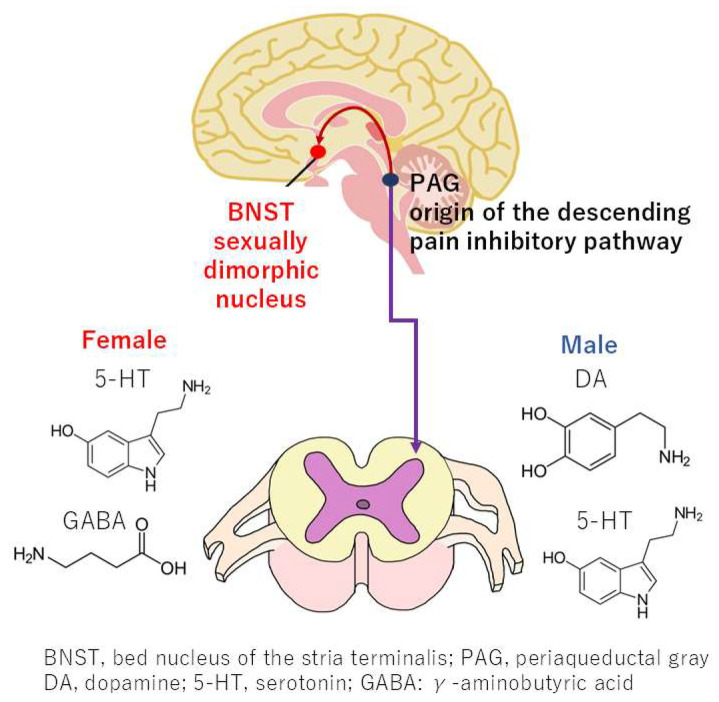
Sex differences in neurotransmitters within the descending pain inhibitory pathway and sexually dimorphic nuclei in the bed nucleus of the stria terminalis.

The expression of neurotransmitters in the nervous system that activate descending pain inhibitory pathways differs between women and men [21,22]. The descending pain inhibitory pathway constitutes a nervous system that alleviates pain through the action of nerves descending from the brainstem to the spinal cord. The origin of this pathway is the periaqueductal gray (PAG), a midbrain structure that plays a pivotal role in regulating pain. The detection of pain by the cerebral cortex leads to the release of dopamine from the ventral tegmental area, which activates the PAG and transmits signals to the bed nucleus of stria terminalis (BNST). Sex differences in the structure of the BNST have been observed, with women exhibiting a heightened tendency toward anxiety [21] (Figure 4). Preclinical studies have demonstrated that, under specific conditions, the transmission of stimuli from the PAG to the spinal cord results in the release of serotonin and dopamine in male rats and serotonin and GABA in female rats [22]. This system has been implicated in both the regulation of pain and the defecation reflex. However, given the differential expression of these monoamines, the defecation reflex is observed to be less pronounced in women compared to men [23]. While the underlying mechanisms remain to be fully elucidated, emerging evidence suggests that there are differences between men and women in the expression of monoamine receptors in descending pain inhibitory pathways and associated neural systems. These variations are more likely to contribute to the transmission of pain and the induction of anxiety in women [21].

## 5. Sex Differences in the Brain Network Pain Circuit

### 5.1. Large-Scale Brain Networks

The predominant hypothesis posits that brain network pain, an aberration in the network of neural regions responsible for the perception of pain, accounts for the majority of nociplastic pain and is one of the primary mechanisms of BMS [11]. The long-standing hypothesis that specific functions are confined to specific regions of the brain is being challenged by the emerging paradigm that network interactions are pivotal to comprehending pain. The study of these networks is undergoing a paradigm shift, driven by advancements in brain imaging techniques such as functional magnetic resonance imaging (fMRI) and analytical tools such as graph theory. These tools provide a framework for understanding how the brain functions as an integrated entity, rather than as a collection of discrete components. Large-scale brain networks provide a more comprehensive and dynamic perspective on brain function, playing a crucial role in comprehending normal cognition as well as pathological conditions, such as pain perception [24]. These networks provide a more accurate model of cognitive functions, such as attention and pain perception, by considering the interaction of multiple brain regions [25]. Research findings have revealed substantial disparities in brain networks between males and females, including alterations in connectivity, hierarchy, and interaction patterns. These disparities may contribute to observed sex differences in pain perception. Preclinical studies in rats have demonstrated that older females may exhibit heightened vulnerability to chronic pain, attributable to alterations in neural connectivity within the brain [26].

### 5.2. Changes in Functional Connectivity of the Default Mode Network

**Figure 5:**
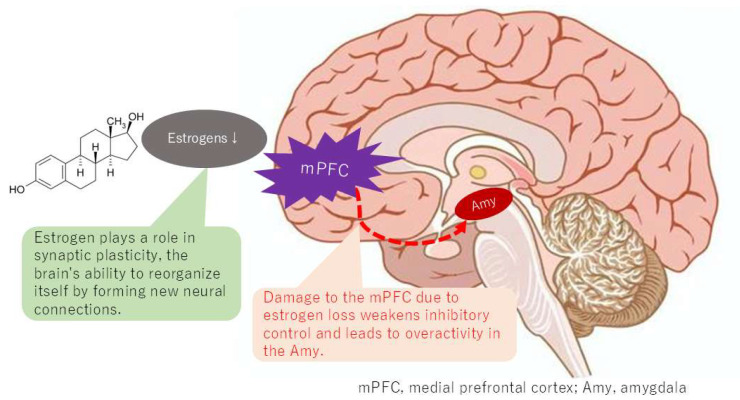
Changes in connectivity of the default mode network with estrogen depletion.

A substantial body of research employing fMRI to compare the brains of individuals with BMS and healthy controls has revealed reduced gray matter volume in the medial prefrontal cortex (mPFC) and altered connectivity of the default mode network (DMN) [27]. Gray matter volume is a quantitative metric representing the total amount of gray matter within a specific brain region, providing insights into the overall size of the region and its association with neuronal connectivity. The observed reductions in gray matter volume within the mPFC of individuals with BMS have been demonstrated to be indicative of altered connectivity within the DMN and have been associated with heightened pain intensity [28]. While these studies have compared sex- and age-matched BMS patients with healthy controls, the majority of studies have primarily focused on female cases. Therefore, it is plausible that DMN alterations may be more prevalent in females with BMS (Figure 5).

The DMN has been demonstrated to comprise a set of regions that exhibit heightened levels of activity during passive tasks relative to tasks that demand focused external attention [29]. A notable observation in the literature is the stronger functional connectivity within the DMN, which includes the posterior cingulate cortex, precuneus, and mPFC, exhibited by younger females. In contrast, adolescent boys and girls have been shown to exhibit divergent profiles of age-related changes in the connectivity of the DMN [30]. The menopausal transition, characterized by a significant decline in estrogen levels, has been associated with alterations in DMN connectivity. Estrogen, known for its neuroprotective properties, can aid in preserving brain cells [31]. This neuroprotection extends to regions within the DMN, influencing synaptic plasticity and the brain’s capacity for self-organization through the formation of new neural connections [32]. This process is crucial for optimal DMN function. The use of fMRI has revealed that estrogen levels can influence the strength and patterns of connectivity within the DMN [33]. Dynamic changes have been demonstrated to involve a reorganization of the DMN depending on estrogen levels [34]. A potential explanation for the higher prevalence of BMS in women may be the alteration of connectivity in the DMN caused by estrogen levels, which could result in a lower threshold for pain perception compared to men.

Individuals diagnosed with BMS demonstrate aberrations in the typical communication patterns within the DMN, which can manifest as both augmented and diminished connectivity between disparate DMN regions. Specifically, there are alterations in the connectivity between the mPFC and other DMN areas, as well as with other brain regions involved in pain and emotional processing, such as the amygdala. The influence of estrogen on brain regions that are part of the DMN, including the mPFC, posterior cingulate cortex, and precuneus, has been demonstrated. Estrogen plays a role in synaptic plasticity, the brain’s ability to reorganize itself by forming new neural connections; a process that is crucial for the proper functioning of the DMN. Estrogen has neuroprotective properties, helping to maintain the health and integrity of brain cells within the DMN and other brain regions. This suggests that estrogen levels can influence the efficiency of communication between different components of the DMN. However, it is crucial to acknowledge that the specific patterns of connectivity alterations within the DMN can vary among individuals and across studies, reflecting the complexity of BMS and the influence of factors such as symptom duration and individual differences.

## 6. Sex Differences in the Memorized Pain Circuit

Individuals afflicted with bruxism are more prone to develop oral habits, such as teeth grinding and jaw clenching. Furthermore, these individuals frequently possess a medical history marked by painful dental procedures that stimulate the trigeminal nerve [35]. This history of nociceptive input from the trigeminal region may serve as a trigger for the onset of BMS. Moreover, the experience of pain during dental procedures has been demonstrated to induce feelings of anxiety and fear, which have been shown to intensify pain perception and contribute to the development of chronic pain disorders [36]. It is important to note that the hippocampus is not the sole locus for memory storage. Recent preclinical studies employing activity-dependent cell labeling and chemogenetic reactivation have demonstrated that neurons in the anterior insular cortex retain an immunological memory of painful inflammation [36]. The activation of neurons in the anterior insular cortex, even after the initial noxious stimulation has subsided, has been shown to reproduce the immunological information stored in these cells. This phenomenon results in the re-emergence of inflammation and pain in the same location as the original stimulation. The hypothesis suggests that memorized pain is caused by the location and inflammatory information being stored in specific brain cells, such as the anterior insular cortex, as an immunological memory. This memory is hypothesized to be reproduced when these cells become excited by emotions or other factors. 

A comparison of the brains of patients with BMS with those of healthy controls reveals higher activation in the anterior insula and anterior cingulate cortex, components of the salience network (SN) [37]. Additionally, strong functional connectivity between the hippocampus and amygdala in BMS patients has been demonstrated within this network [38]. The activation of the anterior insula, in conjunction with the enhancement of its functional connections with the hippocampus and amygdala, may signify a brain mechanism that contributes to the reproduction of memorized pain, the induction of anxiety and depression, and the production of distress in BMS patients [39].

**Figure 6:**
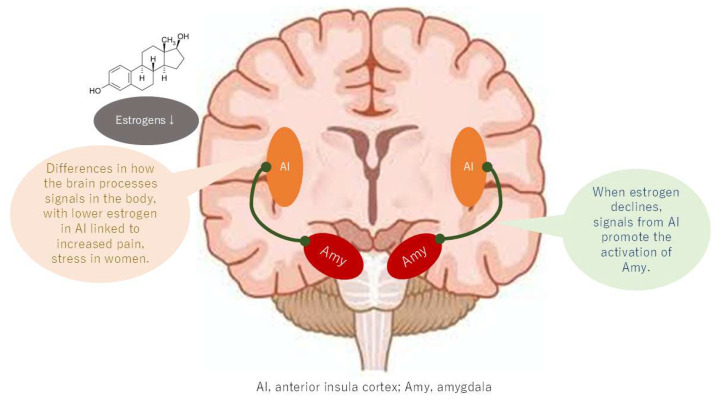
Changes in connectivity of the salience network with estrogen depletion.

Recent research has identified structural and functional sex differences in the anterior insula that are influenced by sex hormones [40]. These findings may be relevant to understanding sex differences in chronic pain. Furthermore, the resting-state functional connectivity of the temporoparietal junction and inferior frontal gyrus of the insular cortex exhibits sex-related differences in women, with higher empathy and stronger personal distress predicted by these differences. This finding suggests that the insular cortex’s sex-related functional differences may render women more susceptible to the memorized pain (Figure 6). Furthermore, insular cortex activation, which occurs due to decreased function of the DMN, suggests that sex differences in the DMN may indirectly contribute to memorized pain being more likely in women.

## 7. Levels of Evidence for Estrogen-Mediated Mechanisms

Mechanisms by which estrogen may be involved in pain generation in the peripheral pain circuit include control of TRPV1, changes in NMDA receptor sensitivity, cell formation in the BNST, and control of neurotransmitter expression in the descending pain inhibitory pathway. However, these are all preclinical. In contrast, studies of brain network pain caused by reduced mPFC function and changes in DMN connectivity, and memorized pain caused by activation of the anterior insula and anterior cingulate gyrus are both clinical studies using fMRI. Representative studies of each are shown in Table 1, and the level of evidence for the mechanism of pain in BMS is thought to be higher for structural and functional changes in the CNS than for peripheral nerve damage. 

## 8. Sex Differences in ICOP Classification

The ICOP classification is a comprehensive framework that categorizes orofacial pain into six distinct groups, ranging from categories 1 to 6. This classification offers a more nuanced understanding of pain mechanisms by considering the contributions of three distinct pain circuits: the peripheral pain circuit, the brain network pain circuit, and the memorized pain circuit. These circuits are subject to analysis at the site of neural circuit damage, offering a more sophisticated approach to pain research and management. BMS (category 6) is classified as nociplastic pain, indicating alterations in nociception (pain signaling) without overt tissue damage. Neural circuit analysis reveals a minimal contribution of the peripheral pain circuit and a substantial contribution from brain network and memorized pain circuits. This suggests that BMS involves changes in brain network connectivity and activation of pain memory pathways. As the ICOP classification progresses from category 1 to 6, there is a concomitant decrease in the involvement of the peripheral pain circuit and an increase in the involvement of the brain network and memorized pain circuits. This shift indicates a transition from peripheral to central pain mechanisms. It has been observed that the proportion of women increases from categories 1 to 6. Although exact statistics are lacking, the odds ratios of women to men estimated from various reports show a steep increase from category 3 (TMJ pain) to a very high proportion of women in category 6 (BMS). Sex differences in the DMN and SN may contribute to these observed differences in disease prevalence. In essence, this indicates the need to transition from a purely peripheral view of orofacial pain to a more integrative model that incorporates CNS mechanisms and potential sex differences. However, social roles and other non-biological factors could also influence brain circuit formation. Therefore, it is important not to conclude that women are simply physiologically at a higher risk of pain.

## 9. Treatment

### 9.1. Treatment for the Peripheral Pain Circuit

Some oral rinses containing mild numbing agents, such as lidocaine, have been shown to provide temporary relief for peripheral pain associated with BMS. Capsaicin, a compound found in chili peppers, has been employed topically to induce desensitization in the affected area [41]. The administration of topical clonazepam rinses or dissolvable wafers has been recommended in cases of burning sensations [42]. However, the efficacy of topical gabapentin solution for the management of burning mouth syndrome has been found to be minimal [43]. While oral pain sensations may vary slightly between sexes, the effectiveness of topical treatments exhibits minimal sex-related differences, is not sex-specific, and may be influenced by a variety of individual factors, including psychological ones [41,42,43].

### 9.2. Treatment for the Brain Network Pain Circuit and Memorized Pain Circuit

Two treatment modalities employed in the management of brain network pain and memorized pain are pharmacotherapy and cognitive behavioral therapy (CBT). The pharmaceutical interventions encompass medications such as clonazepam, antidepressants like amitriptyline, and alpha-lipoic acid. Of these, clonazepam has demonstrated efficacy in randomized controlled trials; however, its effect size is modest [44]. A multitude of studies examining BMS treatments are encumbered by limitations, including diminutive sample sizes, variations in study design, and disparate outcome measures. The complexity of modulating the connections throughout the entire brain by regulating monoamines is another factor contributing to the limited efficacy of drug therapy.

The CBT has demonstrated efficacy when utilized in conjunction with pharmaceutical interventions [45]. CBT exerts a regulatory effect on the function of the DMN, suggesting its potential for addressing brain network-related pain. The efficacy of transcranial magnetic stimulation (TMS) treatments can exhibit considerable variability among individuals, underscoring the necessity for treatments that take into account the unique characteristics of brain networks. In this regard, research into visualizing brain networks is imperative. While fMRI is the prevailing method for evaluating the DMN, it necessitates substantial financial resources and specialized equipment. Functional near-infrared spectroscopy (fNIRS) is an alternative method for detecting the DMN in children, in cases where fMRI is not feasible [46]. Although fNIRS has a lower spatial resolution, it is relatively inexpensive and has enabled real-time detection of the DMN. The analysis of these devices using machine learning may lead to the development of customized treatments for individual BMS patients. To this end, research into the mechanism by which estrogen affects brain circuit formation is also necessary.

### 9.3. Hormone Replacement Therapy

BMS is most prevalent in women going through menopause, and it has been speculated that hormonal fluctuations may alter the nervous system responsible for pain. This hypothesis posits that the decline in neuroprotective gonadal hormones, concomitant with an escalation in stress hormone levels–characteristics of the menopausal transition–serves as a catalyst for this process. Despite the established correlation between BMS and menopause, hormone replacement therapy (HRT) has demonstrated limited efficacy in addressing BMS. A substantial body of research has demonstrated the ineffectiveness of HRT in addressing BMS in postmenopausal women [47]. It is speculated that years of hormonal fluctuations have affected the formation of the nervous system, so simply replacing hormones will not alleviate pain. Given the nociplastic nature of BMS and its distinct etiology from menopause, it is implausible that HRT will exert a direct effect on its alleviation.

## 10. Limitations and Future Challenges

The marked female predominance and menopausal age at which BMS occurs strongly suggest the involvement of hormonal factors [48]. In this paper, we explore, from a neural circuit perspective, whether estrogen fluctuations may be a mechanism underlying sex differences in the prevalence of BMS. The conflicting estrogen levels observed in individuals with BMS suggest a potentially biphasic effect, where estrogen can both promote and inhibit pain [49]. This complexity poses challenges in formulating straightforward conclusions. Genetic factors have been identified as contributors to an individual’s susceptibility to BMS and their response to hormonal changes. Psychosocial factors, such as stress and anxiety, have been demonstrated to influence pain perception and contribute to the development and maintenance of chronic pain. These factors must be considered when conducting research on BMS. Consequently, further research is imperative to elucidate the intricate interplay of these factors and to develop effective treatment strategies. 

It has been established that biological males and females exhibit divergent perceptions of pain, a phenomenon that may be partially explained by their distinct hormonal profiles since birth, which are further amplified during puberty. The complex interplay among hormones, brain regions, and immune responses collectively mediates the sexual dimorphism in pain perception. For biological males, elevated testosterone levels have been demonstrated to augment their pain threshold, while for biological females, fluctuations in estrogen have been shown to enhance pain intensity and perception [50]. However, it is imperative to acknowledge that this does not paint the full picture. Sexual dimorphism does not imply that females are inherently at a disadvantage. It has been demonstrated that women may exhibit enhanced adaptation and habituation to persistent heat pain. This adaptation may be attributed to the potential for greater habituation to persistent heat pain in women [51]. Voluntary exercise, such as running, has been shown to effectively mitigate nociceptive stimuli in females by inhibiting inflammatory responses and dorsal root ganglion (DRG) excitability. Elucidation of these mechanisms may provide avenues for the alleviation of women’s experiences with chronic pain. This study aims to contribute to the understanding of sex differences in brain function by examining the role of estrogen in the formation of functional connections in the DMN and SN. Regarding neurotransmitters, evidence suggests potential sex differences in opioids, serotonin, and dopamine; however, further elucidation is necessary to fully clarify these matters. It is imperative to consider the potential influence of genetic and psychosocial factors on these variations, given the recognized disparities in cultural norms, gender roles, and stress between the sexes. Addressing these points will facilitate a more comprehensive and informative discussion on sex differences in pain perception.

## 11. Conclusions

BMS is a chronic pain disorder of unknown etiology that is prevalent among women. The pain associated with BMS is classified as nociplastic pain, that includes the peripheral pain circuit, brain network pain circuit, and memorized pain circuit. In the context of peripheral pain circuit, estrogen has been demonstrated to play a role in the expression of TRPV1 receptors. In the context of brain network pain circuit, fluctuations in estrogen levels have been observed to impact the function and connectivity of the DMN. The anterior insular cortex is also activated by decreased function of the DMN, and an indirect sex difference is observed due to the influence of the DMN on brain circuits. Consequently, the development of BMS may be influenced by the formation of neural circuits by sex hormones. Given the heterogeneity of individuals with BMS and their poor response to pharmacotherapy, combination treatments that target the DMN, such as cognitive behavioral therapy, are imperative.

## Figures and Tables

**Table 1:** Estrogen-mediated mechanisms in the three neural circuits of burning mouth syndrome.

Author (Year)	Pain Circuit	Estrogen-Mediated Mechanisms	Study Phase
Seol et al., 2022 [15]	Peripheral pain	Regulation of expression and intracellular trafficking of transient receptor potential cation channel subfamily V member 1 (TRPV1)	Preclinical
Taubøll et al., 2015 [18]	Peripheral pain	Changes in the sensitivity of N-Methyl-D-aspartic acid (NMDA) receptors and are involved in the wind-up phenomenon	Preclinical
Shen et al., 2023 [21]	Peripheral pain	Affects the structure of the bed nucleus of the stria terminalis (BNST) and alters emotional responses	Preclinical
Horii et al., 2023 [23]	Peripheral pain	Regulation of neurotransmitter expression in the descending pain inhibitory pathway	Preclinical
Ong et al., 2019 [28]	Brain network pain	Hypofunction of the medial prefrontal cortex (mPFC) and altered connectivity of the default mode network	Clinical
McBenedict et al., 2024 [39]	Memorized pain	Involved in activation of the anterior insular cortex and anterior cingulate cortex	Clinical

## Data Availability

The data that support the findings of this study are available from the corresponding author upon reasonable request.

## Abbreviations

The following abbreviations are used in this manuscript:

BMS	burning mouth syndrome
BNST	bed nucleus of stria terminalis
CBT	cognitive behavioral therapy
DMN	default mode network
fMRI	functional magnetic resonance imaging
fNIRS	functional near-infrared spectroscopy
HRT	hormone replacement therapy
IASP	International Association for the Study of Pain
ICOP	International Classification of Orofacial Pain
mPFC	medial prefrontal cortex
NGF	nerve growth factor
NMDA	N-methyl-D-aspartate
PAG	periaqueductal gray
PNS	peripheral nervous system
SN	salience network
TMS	transcranial magnetic stimulation
TRPV1	transient receptor potential cation channel subfamily vanilloid member 1
